# Effectiveness of Neurodevelopmental Treatment and Sensory Integration Therapy on Gross Motor Function, Balance and Gait Parameters in Children With Spastic Diplegia

**DOI:** 10.7759/cureus.43876

**Published:** 2023-08-21

**Authors:** Anushka Raipure, Rakesh Krishna Kovela, Pallavi Harjpal

**Affiliations:** 1 Department of Neuro Physiotherapy, Ravi Nair Physiotherapy College, Datta Meghe Institute of Higher Education & Research (Deemed to be University), Wardha, IND; 2 Department of Neuro Physiotherapy, Nitte Institute of Physiotherapy, NITTE (Deemed to be University), Mangaluru, IND

**Keywords:** spastic diplegia, balance, gross motor function, gait parameters, sensory integration therapy, neurodevelopmental treatment

## Abstract

Background

Spastic diplegic cerebral palsy is the type that is most frequently seen in clinical settings. Spastic diplegic children have trouble maintaining their balance, gait, and gross motor function. This study investigated the effects of the Neurodevelopmental Technique (NDT) and Sensory Integration Technique (SIT) on balance, gross motor function, and gait characteristics in children with spastic diplegia.

Method

The study's participants were 8 to 12 years old, with spastic diplegia, categorized into stages I to III of the Gross Motor Function Classification System. While individuals in group B underwent sensory integration therapy, group A's subjects received NDT for 45 minutes. Both groups received traditional physiotherapy for 15 minutes. The protocol was given for five days a week, continuously for four weeks. All 40 subjects underwent pre- and post-treatment assessments using the Gross Motor Function Measure-88 (GMFM-88), Paediatric Balance Scale, Gait Parameters, and Gross Motor Function Classification System.

Results

The trial involved 40 children, divided into two groups of 20 each. Statistical analysis demonstrated a substantial improvement in group B post-intervention (P>0.0001). The study's findings were drawn using the Chi-Square test, paired and unpaired t-tests, and SPSS Statistics for Windows, version 27.0 (IBM Corp., Armonk, USA).A p<0.05 and the GraphPad Prism version 7.0 (GraphPad Software, Boston, USA) were used*. *A total of 40 children completed the entire duration of treatment for a month. 20 subjects participated in group A (age range 8-12 years; mean age 10.3 years) and 20 subjects in group B (age range 8-12 years; mean age 10.25 years). The GMFM-88, which assesses motor function, reveals that the between-group comparison indicates a substantial difference of 7.95 (6.04-9.86) in favor of Group B, with a p-value of 0.0001, signifying statistical significance. Similarly, the Pediatric Balance Scale (PBS) outcomes significantly enhanced in both groups post-intervention. The comparison between groups yields a difference of 1.85 (1.11-2.59) in favor of Group B, with a p-value of 0.0001.

Conclusion

The study concluded that SIT has a positive impact on gait metrics, balance, and gross motor function in children with spastic diplegia.

## Introduction

"Cerebral palsy" or static encephalopathy refers to a wide range of cognitive and motor problems brought on by disturbance to the maturing central nervous system (CNS) [1]. It incorporates a variety of motor dysfunction syndromes that are non-progressive but typically changing, caused by injuries or anomalies of the brain during early developmental stages [2]. Together with the motor abnormalities of cerebral palsy, secondary musculoskeletal problems, seizures, and modifications in behaviour are frequently present [3].

In spastic diplegia, there is little to no involvement of the head, neck, or trunk, and the lower limbs are the primary areas affected by spasticity. Periventricular leukomalacia, the primary site of spastic diplegic cerebral palsy, is discernible on magnetic resonance imaging [4]. Cerebral palsy (CP) is classified into the following groups based on the affected extremities and signs of neurologic impairment. The primary classifications encompass spastic CP, characterized by elevated muscle tone and hypertonia; dyskinetic (athetoid) CP, delineated by uncontrolled, involuntary writhing movements; and ataxic CP, denoted by cerebellar dysfunction resulting in disruptions of coordination and balance. The location of lesions, the underlying disease, the chronologic age, and the gestational age at delivery all affect the clinical symptoms differently [5]. Congenital, inflammatory, viral, traumatic, and metabolic factors are the aetiologic factors of cerebral palsy [6]. Prenatal damage, which encompasses adverse developmental effects during the gestational period, influences a considerable demographic of patients, containing an estimated range of 75% to 80% of cases. In a distinct albeit less prevalent scenario, a subset comprising approximately 5% to 10% of instances encounters the aftermath of profound birth trauma or hypoxia, both of which signify critical complications arising during the labor and delivery process [7]. Cerebral palsy affects 10% to 18% of newborns who weigh 500 to 999 grams [8].

Upper motor neuron symptoms, weakness, hypertonia, and hyperreflexia are all seen in spastic types. Extrapyramidal involvement is evident in dyskinetic CP, resulting in stiffness, chorea, and dystonic movements. In CP, primitive reflexes become more pronounced and last longer. During sleep, such movement patterns are eliminated, and the tone of the afflicted limbs decreases. Abnormalities in posture control and coordination are also present. Hypotonic children at birth may evolve into this type by one to three years old. There are no cognitive deficits in most of this class [9]. There are numerous techniques now being utilised to treat childhood CP that seem to have the ability to enhance motor skills. Children with cerebral palsy have benefited from neurodevelopmental therapy and sensory integration therapy since their inception [10].

In the 1970s, Jean Ayres coined the phrase "sensory integration therapy" (SIT). Information processing is the integration of the senses. The brain must pick out sensory information, enhance, inhibit, compare, and associate in a fluid, ever-changing pattern or integrated. The SIT approach supports a child's normal development by improving their ability to receive and process sensory data [11]. It is a theory and neurological procedure that allow a person to organise and produce structured action by analysing, incorporating, and using the spatiotemporal components from the environment. It contends that for children to learn, they must be able to interpret sensory input from their surroundings, internalise these impulses throughout the CNS, and employ this information to produce ordered behaviour [12].

In 1985, Ayres wrote that praxis is the mechanism by which cognition drives motor activity; motor planning is the transitional phase which blends conceptualization and motor execution to enable appropriate exchanges with the environment. Praxis can be simply described as "will-based activity". It includes the method of conceiving and organizing such motor actions. It is a procedure that calls for the person to have knowledge of actions and objects, motivation, and intention [13]. Ideation (conceptualizing activities), motor planning, and implementation comprise Praxis, one of Ayres' key concepts. Ayres has consistently stressed the importance of visual and somatic awareness in practice, as seen in her early works [14]. 

Dyspraxia is described as having trouble sequencing and planning skills and non-habitual motor acts. According to Ayres, somatodyspraxia is a deficit of imprinting a novel motor response approach instead of a familiar one. All dyspraxic clients experience a motor planning weakness, leading to motor clumsiness. Due to inadequate vestibular and proprioceptive processing, the youngster would also have trouble timing and sequencing movements. The child would have inappropriate body plans because of insufficient motor abilities [15].

In order to approach the central nervous system and to improve and achieve motor performance in CP, the NDT approach is the most well-liked method. This tactic's main objective is to normalise movement patterns by addressing abnormal postural tones [16]. Following passive stretching of the muscles in the lower limbs (such as the gastrosoleus and hamstrings), techniques for reducing spasticity and allowing more natural movement patterns are employed while concentrating on the motor processes. These therapeutic goals (normalising the movement) will be attained through direct physical contact with the child during movement, offering them more conventional sensorimotor exposures. Handling techniques and therapeutic activities remain dynamic, constantly adapting to suit the individual responses exhibited by each child [17].

While some studies suggest that NDT is the most effective therapy for children with spastic diplegia, others found that SIT can also be utilised to improve motor function since CP children may have sensory impairment owing to Brain injury, which may subsequently lead to motor dysfunction [18]. This study aimed to investigate how sensory integration therapy affects balance, walking, and other gross motor skills. The study examined how gait, balance, and gross motor performance were affected by sensory integration therapy and neurodevelopmental therapy. The study also sheds light on how cerebral palsy patients are impacted by SIT. This study clarifies the benefits of sensory inputs for motor learning and their incorporation into the training process. The study will also make it possible to create a more effective treatment plan for children with spastic diplegic disorders.

## Materials and methods

The study was carried out at the Acharya Vinoba Bhave Rural Hospital in Sawangi, Meghe, Wardha, Maharashtra, at the neuro physiotherapy outpatient department (OPD). This was carried out after ethical clearance from the Datta Meghe Institute of Higher Education and Research Institutional Ethical Committee (Ethical authorization number: DMIMS [DU]/IEC/2022/1002). 

The trial involved 40 children, strategically allocated into two discrete groups, each encompassing a membership of 20 children. We initiated this partitioning to establish a well-balanced distribution of participants, guaranteeing the representation of each group within the broader population under study. After completing the trial, we conducted a detailed statistical analysis to scrutinize the outcomes. Notably, the analytical examination revealed a conspicuous advancement in group B's post-intervention state of affairs. This advancement was characterized by a discernible amelioration in the studied parameters, indicating a meaningful and substantial positive effect resulting from the intervention. Researchers quantified the statistical significance of this improvement by calculating a p-value, a metric used in hypothesis testing to determine the probability of observing such a significant enhancement solely as a result of chance. In this particular instance, we decided that the calculated p-value was below 0.0001, signifying an exceedingly low likelihood that the observed enhancement in group B could be attributed to random variations. Hence, this trial's outcomes emphasize the intervention's effectiveness and influence, supported by the statistically substantial and noteworthy progress witnessed within group B. This outcome contributes to the growing body of evidence supporting the potential benefits of this intervention in the context of the studied parameters and the target population.

**Figure 1:**
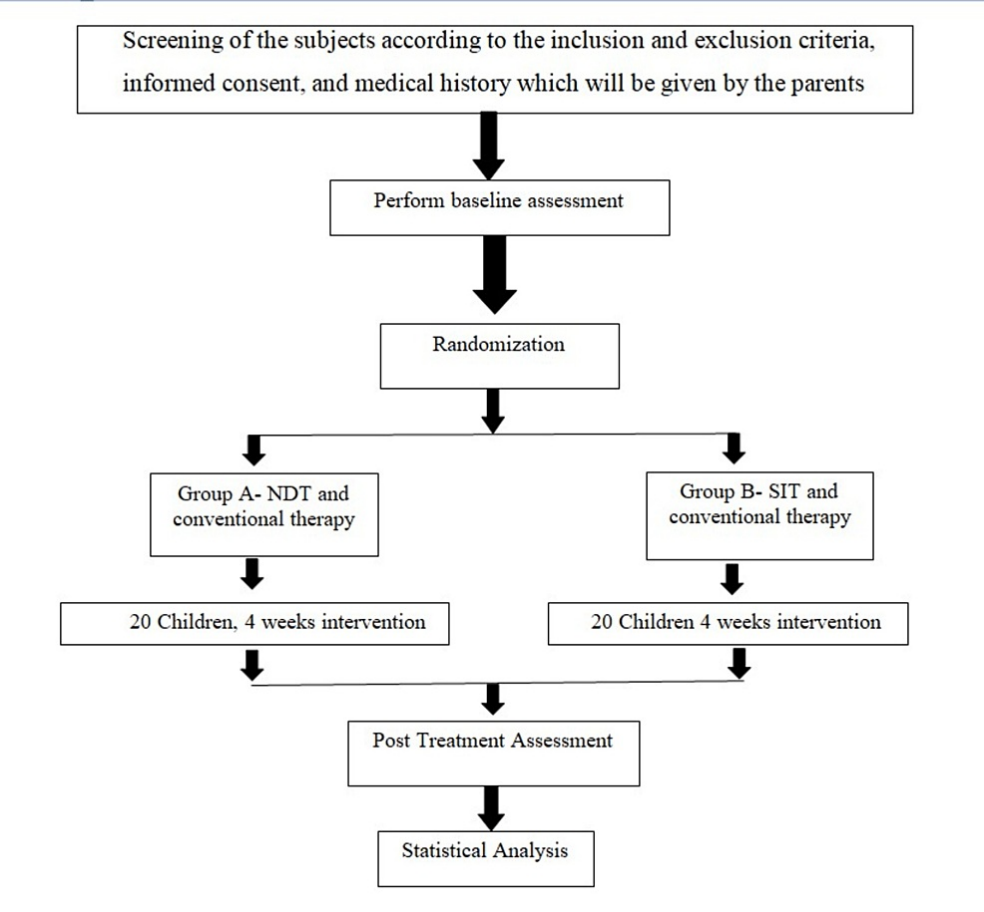
Flowchart of the study procedure. NDT: Neurodevelopmental Technique; SIT: Sensory Integration Technique

Before beginning therapy, the participants had to sign consent documents that the researcher had provided. Before obtaining their individual forms, all participants were given instructions on how to complete them. The Gross Motor Motor Function Measure-88 (GMFM-88), Paediatric Balance Scale (PBS), Gait Parameters, and Gross Motor Function Classification Scale (GMFCS) were used to collect pre-treatment or baseline data on each patient following the completion of consent forms. Age between 8 and 12 years, independence in sitting and walking, GMFCS levels I to III, and a score of greater than 22 on the Mini-Mental Status Assessment were the inclusion criteria. Children with levels IV and V of the GMFCS, and children who had uncontrolled seizures or surgery within the previous six months, were all excluded from the study. Based on the inclusion criteria, 40 patients were picked for the study. The participants were picked at random and put into groups A and B. The methodology flowchart for the study is shown in Figure 1. 

Outcome measures

Gross Motor Function Measure-88 (GMFM-88)

The instrument is now used extensively around the world as the benchmark outcome monitoring instrument for treatment interventions in cerebral palsy. Based on gross motor function, the 88 items in this clinical examination are separated into five categories. Children are observed while they complete the 88 items on the GMFM, which is then scored on a 4-point ordinal scale [19].

Pediatric Balance Scale (PBS)

It is an evaluation scale with 14 components that can be used to assess the static and dynamic balance of the population. PBS is used in school students between the ages of 5 and 15 years [20].

Gait Parameters

In the current study, gait metrics included the number of steps per minute, stride length (the distance between one lower limb's heel strike and the next), and gait velocity [21].

Gross Motor Function Classification System (GMFCS)

The GMFCS is a straightforward, five-level ordinal grading system designed to describe a cerebral palsy patient's gross motor performance [22].

Intervention

The same outcome measures were used for both assessments of the subjects. Those assigned to group A received NDT for 45 minutes five days a week for four weeks. In contrast, those assigned to group B received SIT for 45 minutes, for the same period.

Both groups of subjects had a 15-minute session of traditional physiotherapy, which comprised passive stretches in both lower limbs. Exercises for passive and active range of motion for the bilateral lower limbs were administered. Children were instructed to take the pegs from the pegboard before them and put them back in.

The test subjects in group A received three NDT components. With the occupational therapists help, children supported themselves with their hands and forearms when sitting, crawling, semi-kneeling, and standing. This helped them develop their gross motor skills. A CP ball were then utilised to encourage balance and corrective reflexes after the children had learned the capacity to hold workout positions. The ambulation training that was provided included crawling, creeping, walking while semi-kneeling, and walking between parallel bars, depending on the student's motor development level [23]. Balance training involved trunk activation in every plane while the patient was sitting or standing. The therapist assisted this with hands-on facilitation. Weight shifts and trunk elongation on the ideal trunk alignment in sitting and standing positions are two NDT concepts applied in these workouts. The child was challenged when standing and grabbing something in the transverse plane. Making the use of the knee, the therapist helped the patient engage their hip joint and provide stability so that their trunk could rotate to reach the target object. Gait training was given using just a small pelvic support help, stepping forward, and reaching out [16].

Subjects in group B were given SIT, including jumping, rolling, sitting and spinning, swinging, dancing, trampoline, and slides. For improving balance, subjects were given weighted objects and heavy work patterns to encourage co-activation and stabilization of the muscle activity. Subjects were also given clay activities, hammering and cutting. Subjects were given riding equipment that required them to mobilize, activating the muscles. Subjects were also given climbing activities using ropes, ladders and ramps. To improve gait, the subjects were given walking and standing training [24].

## Results

The study's findings were drawn using the Chi-Square test, paired and unpaired t-tests, and SPSS Statistics for Windows, version 27.0 (IBM Corp., Armonk, USA). A p<0.05 and the GraphPad Prism version 7.0 (GraphPad Software, Boston, USA) were used. A total of 40 children completed the entire duration of treatment for a month. 20 subjects participated in group A (age range 8-12 years; mean age 10.3 years) and 20 subjects in group B (age range 8-12 years; mean age 10.25 years). Table 1 provides the age group distribution in both groups.

**Table 1:** Participants in both groups according to their respective age groups

Age Group (in years)	Group A	Group B
8	2	3
9	4	3
10	5	4
11	4	6
12	5	4
Mean±SD	10.3±1.307	10.25±1.336
Range	8 to 12	8 to 12

Table 2 presents a comprehensive overview of outcome measures before and after intervention in two distinct groups, group A and group B. Each outcome measure is analyzed and compared between the two groups, with statistical values provided to indicate the significance of the findings.

**Table 2:** Within-group and between-group comparison of all the outcome measures GMFM: Gross Motor Function Measure; PBS: Paediatric Balance Scale; S = significant

Outcome measures	Group A Pre	Group A post	Post-Pre (95% CI)	Group B Pre	Group B Post	Post-Pre (95% CI)	Between-group comparison of Post-test (95% CI)	p-value
GMFM-88	20.20 ± 3.41	47.10 ± 2.34	26.90 (25.44-28.36)	19.75 ± 2.71	55.05 ± 3.50	35.3 (33.31-37.29)	7.95 (6.04-9.86)	0.0001, S
PBS	15.50 ± 2.86	48.40± 0.99	32.90 (31.51-34.29)	16.55± 2.80	50.25 ± 1.29	33.7 (32.44-34.96)	1.85 (1.11-2.59)	0.0001, S
Stride Length (cm)	30.60 ±5.31	45.15 ±0.81	14.55 (12.12-16.98)	27.60 ± 1.82	45.75 ± 0.91	18.15 (17.21-19.09)	0.60 (0.05-1.15)	0.0341, S
Cadence (steps/min)	16.30±0.86	20.45±1.50	4.15 (3.43-4.87)	15.45 ± 1.00	26.80 ± 0.70	11.35 (10.76-11.94)	6.35 (5.60-7.10)	0.0001, S
Gait Velocity (m/sec)	0.3725 ±0.1023	0.7425 ± 0.0606	0.3700 (0.3056-0.4344)	0.3485 ± 0.0868	1.2250 ± 0.2741	0.8765 ( 0.7417-1.0113)	0.4825 (0.3554-0.6096)	0.0001, S

The first outcome measure, GMFM-88, which assesses motor function, reveals that group A displayed an initial mean score of 20.20 ± 3.41, significantly improving to 47.10 ± 2.34 post-intervention. In contrast, group B started at 19.75 ± 2.71 and remarkably improved to 55.05 ± 3.50. The between-group comparison indicates a substantial difference of 7.95 (6.04-9.86) in favor of group B, with a p-value of 0.0001, signifying statistical significance.

Similarly, the Pediatric Balance Scale (PBS) outcomes were significantly enhanced in both groups post-intervention. Group A demonstrated a notable increase from 15.50 ± 2.86 to 48.40 ± 0.99, while group B improved from 16.55 ± 2.80 to 50.25 ± 1.29. The comparison between groups yields a difference of 1.85 (1.11-2.59) in favor of group B, again with a p-value of 0.0001.

Stride length, another measure, indicates an improvement in both groups' walking ability. Group A increased from 30.60 ± 5.31 to 45.15 ± 0.81, and group B improved from 27.60 ± 1.82 to 45.75 ± 0.91. The between-group comparison demonstrates a difference of 0.60 (0.05-1.15) with a p-value of 0.0341.

Cadence, the measure of step rate, increased in both groups after the intervention. Group A went from 16.30 ± 0.86 to 20.45 ± 1.50, while group B increased from 15.45 ± 1.00 to 26.80 ± 0.70. The between-group difference was 6.35 (5.60-7.10) with a p-value of 0.0001.

Gait velocity, representing walking speed, showed improvements in both groups. Group A's velocity changed from 0.3725 ± 0.1023 to 0.7425 ± 0.0606, and group B's velocity increased from 0.3485 ± 0.0868 to 1.2250 ± 0.2741. The between-group difference was 0.4825 (0.3554-0.6096) with a p-value of 0.0001.

This study examined central tendencies and significance levels, employing medians as robust indicators. The summarized statistics table encapsulates pre and post medians for each group. Group A exhibited medians of 5.5 and 5.0, with heightened significance criteria (p < 0.0001) underlining non-significant changes. Similarly, group B demonstrated comparable medians at 5.5 and 5.0, with parallel significance thresholds. This streamlined presentation integrates medians and refined significance, offering a comprehensive insight into dataset dynamics for both groups.

In summary, Table 2 provides a detailed examination of various outcome measures, illustrating the significant improvements observed in most measurements post-intervention for both groups, with careful statistical comparisons emphasizing the significance of these improvements.

## Discussion

Approaches used in occupational therapy and neuro-rehabilitation are crucial parts of the management for children with CP. One of the main objectives of treatment for CP children is gross motor function improvement. The results of this study show that providing children with spastic diplegia with Sensory Integration Therapy enhances their balance, gait metrics, and gross motor function. In light of our findings, it becomes evident that Sensory Integration Therapy (SIT) is notably more effective. Our study's outcomes consistently indicate substantial improvements in various outcome measures, particularly within the SIT group. The statistically significant advancements observed in motor function, balance, stride length, cadence, gait velocity, and gross motor function underscore the robust impact of the Sit-and-Reach intervention. This collective evidence suggests that the SIT protocol holds promise as a potent strategy for enhancing multiple aspects of physical performance. However, further research and direct comparisons with alternative interventions are warranted to understand its comparative effectiveness and applicability across diverse populations comprehensively.

To the best of our knowledge, this is the first study to investigate the effects of NDT and SIT on the gait metrics, balance, and gross motor function of children with cerebral palsy. Many occupational therapy approaches have been used to assist the motor growth of children with cerebral palsy, avoid postural problems, sensory responses, and gross motor dysfunction, and improve their functional capacity. One of the key therapeutic goals for children with CP is the improvement of gross motor function. Children with spastic diplegia participated in this trial and underwent two therapies for one month, which dramatically improved their GMFM-88-measured gross motor function. According to our research, both groups' gross motor skills considerably increased after receiving SIT and NDT. Rolling, sitting, and kneeling greatly improved after neurodevelopmental intervention, according to studies by Ketelaar et al. [25]. These outcomes were in line with what our research showed, which was that NDT intervention led to significant modifications. In a different study, Fetters and Kluzik found that cerebral palsy neurodevelopmental therapy improved the motor skills of children [26]. Only a few researchers have studied the influence of SIT on gross motor function over time. Participants were allocated and got 2 hours of therapy each week for six weeks in Carlsen's randomised controlled experiment. This intervention's duration is nearly identical to our trials. The SIT group's gross motor skills improved noticeably more than those of the control group [27].

According to our study's comparison, gross motor function significantly improved over the course of a month of SIT and NDT treatment. Programs for sensory integration treatment have reportedly been utilised to support motor functioning, according to published literature. SIT and NDT may result in a variety of results with regard to modifications to motor function. The SIT approach helps children develop normally and improves their ability to process and integrate sensory information [28]. The use of programs for SIT to promote motor functioning has been documented in published literature. The SIT approach promotes healthy development while boosting the child's ability to process and integrate information [29]. The SIT technique necessitates that the child's motivation be a major factor in choosing the activities [30]. A functional rehabilitation programme may enhance everyday activities for kids with cerebral palsy while simultaneously enhancing gross motor function, according to the findings of a study by Akbari et al., in which the subjects' gross motor abilities were assessed using the GMFM [31].

Group B demonstrated a more noticeable improvement in balance as compared to group A. By selecting activities that considered dynamic stability, trunk elongation through assistance by guiding hands-on approach, and other factors, the balance of the group B participants improved. According to Sah et al., a 6-week experiment utilising NDT principles improved trunk control, balance, and gross motor function in diplegic CP patients. This was the outcome of a clinical study that was single-blinded and randomised. In children with spastic diplegia, task-oriented NDT exercises are more effective at enhancing the same parameters which has therapeutic implications [16]. Batool et al. investigated in a pilot study whether cerebral palsy patients would benefit from sensory integration treatment (SIT) combined with virtual reality (VR) and traditional physical therapy. They came to the conclusion that, in comparison to virtual reality, adding virtual reality to sensory integration therapy had significant impacts on improving cerebral palsy patients' gross motor skills, balance, and mobility [32].

According to the findings of our study, introducing SIT significantly improved the gait characteristics in kids with spastic CP. SIT's principal objectives are to provide children with a rich sensory experience and to encourage deliberate motor output. It is crucial to employ several sensory data because different balance disturbances engage other senses, and each sensor's sensitivity range frequently varies. Information misrepresented to the CNS by one or more systems can be compensated for by inputs from the other systems. Seyam et al. looked at how well the sensory integration program affected children with hemiplegic cerebral palsy's walking patterns. They found that children demonstrated statistically significant variations in their gait measurements after receiving sensory integration and a traditional physical treatment programme for three months [33]. NDT and gait features in children with spastic diplegia were studied by Malawade and Subhash. They came to the conclusion that applying the neurodevelopmental method improved gait metrics in the spastic diplegic population [34].

This study shows that SIT improves gait characteristics, balance, and gross motor function more effectively than NDT, as indicated by the abovementioned outcomes. Nevertheless, NDT or SIT techniques did not significantly vary in our study's evaluation of GMFCS levels before and after the interventions. This study demonstrated that SIT and NDT enhanced gross motor function. 

In light of our findings, it becomes evident that the Sensory Integration Therapy (SIT) is notably more effective. Our study's outcomes consistently indicate substantial improvements in various outcome measures, particularly within the SIT group. The statistically significant advancements observed in motor function, balance, stride length, cadence, gait velocity, and gross motor function underscore the robust impact of the Sit-and-Reach intervention. This collective evidence suggests that the SIT protocol holds promise as a potent strategy for enhancing multiple aspects of physical performance. However, further research and direct comparisons with alternative interventions are warranted to understand its comparative effectiveness and applicability across diverse populations comprehensively.

## Conclusions

The results of this study have shown that NDT and SIT movements can improve balance, gait, and gross motor function. The results of the study show that NDT and SIT have a promising effect on gait and balance variables, helping the child reach their normal functional potential so they can match with other kids their age in terms of the potencies to carry out different activities. According to this study, SIT positively impacts the parameters' correction.
